# Public Understanding of Wastewater Surveillance, United States, August 2025

**DOI:** 10.3201/eid3213.260559

**Published:** 2026-08

**Authors:** Rieza H. Soelaeman, Diana Valencia, Jena Losch, Danielle Kleven, Jazmyn Moore, Scott Santibañez

**Affiliations:** Centers for Disease Control and Prevention, Atlanta, Georgia, USA

**Keywords:** wastewater surveillance, infectious diseases, surveillance, epidemiology, public health, public opinion, data literacy, United States

## Abstract

We examined public understanding of wastewater surveillance (WS) data and perceptions of its importance by using a nationwide cross-sectional survey. Approximately half of participants were aware of WS, and 79.0% responded that WS information about viruses was important, highlighting the need for community engagement about WS use and value.

The United States National Wastewater Surveillance System, established in 2020, provides public access to timely data about infectious diseases that might be circulating in a community (*1*). Clinicians, public health officials, and the general public can use this information to guide action (*2*,*3*). Despite broad implementation, less is known about how the public interprets wastewater surveillance (WS) data or perceives its value (*4*–*6*). We examined public understanding of WS data and perceptions of its importance.

We collected data by using the PN View survey (https://styles.porternovelli.com/pn-view-panels), a nationwide cross-sectional online survey conducted by Porter Novelli Public Services and Big Village. The voluntary survey was administered from August 22–24, 2025, to adults >18 years of age in the United States sampled by using nonprobability quota sampling. Porter Novelli computed survey weights by using a proprietary process to reflect the United States population demographic composition in sex, age, region, race and ethnicity, and education on the basis of Current Population Survey proportions (Appendix Table). We followed the Checklist for Reporting Results of Internet E-Surveys guidelines (*7*). 

Participants were asked to select the definition of WS that best matched their understanding, interpret a figure depicting wastewater detections of a hypothetical vaccine-preventable respiratory virus, and identify actions they would take if the virus was detected in their community. We collected participants’ opinions on the importance of the information provided by WS by using an open-text field (Appendix). We performed weighted quantitative analyses in Stata version 18.0 (StataCorp, LLC, https://www.stata.com); we analyzed free-text responses in MAXQDA 24 (https://www.maxqda.com/new-maxqda-24). 

This activity was reviewed and deemed not research by the National Center for Emerging and Zoonotic Infectious Diseases Human Subjects Advisor at the Centers for Disease Control and Prevention (CDC) and conducted according to CDC Human Research Protection Program Standard Operating Procedures, consistent with applicable federal law and CDC policy. Although Porter Novelli Public Services and Big Village did not require Institutional Review Board review, they adhere to European Society for Opinion and Marketing Research ethical standards.

Of 1,014 participants, 51.0% (95% CI 47.8%–54.2%) were female and 48.5% (95% CI 45.3–51.7) male; 60.8% (95% CI 57.5%–63.9%) were non-Hispanic White, and 45.9% (95% CI 42.7%–49.1%) lived in a suburban community (Table). Nearly half of all participants (50.3% [95% CI 47.1%–53.5%]) defined WS correctly, and 83.3% (95% CI 80.8%–85.5%) of participants interpreted the figure correctly. The most common actions selected by participants were to learn more about the virus and how it spreads (51.1% [95% CI 47.9%–54.3%]), check CDC’s website for more information (49.1% [95% CI 45.9%–52.3%]), and consult the local health department (45.7% [95% CI 42.5%–48.9%]) (Figure; Appendix). 

**Table:** Public understanding of wastewater surveillance and interpretation of wastewater detection data visualization by demographic characteristic, Porter Novelli View survey, United States, August 2025

Characteristic	No. (%) [95% CI]*	Correctly defined wastewater surveillance, % (95% CI)		p value†	Correctly interpreted the data visualization figure, % (95% CI)		p value†
		Yes, n = 508	No, n = 506		Yes, n = 838	No, n = 176	
Total	1,014 (100.0)	100.0	100.0		100.0	100.0	
Sex				0.53			0.56
M	498 (48.5) [45.3–51.7]	49.9 (45.3–54.4)	47.0 (42.6–51.6)		48.4 (44.9–51.9)	48.8 (41.2–56.5)	
F	506 (51.0) [47.8–54.2]	49.6 (45.0–54.1)	52.5 (48.0–57.0)		51.2 (47.7–54.7)	50.2 (42.5–57.8)	
Missing	10 (0.5) [0.3–0.9]	0.6 (0.3–1.3)	0.4 (0.2–1.1)		0.4 (0.2–0.9)	1.0 (0.3–3.0)	
Age group, y				<0.001			<0.001
18–29	191 (19.1) [16.7–21.8]	16.6 (13.5–20.2)	21.7 (18.1–25.7)		18.0 [15.4–20.9)	24.9 [18.8–32.3)	
30–44	333 (26.7) [24.2–29.4]	22.7 (19.4–26.3)	30.8 (27.0–34.9)		24.6 (22.0–27.6)	37.0 (30.2–44.5)	
45–59	191 (22.8) [20.0–25.8]	23.2 (19.3–27.6)	22.4 (18.6–26.6)		23.6 (20.6–27.0)	18.4 (13.0–25.6)	
>60	299 (31.4) [28.5–34.5]	37.6 (33.2–42.1)	25.1 (21.4–29.4)		33.8 (30.5–37.2)	19.6 (13.9–26.8)	
Race and ethnicity‡							
Black or African American	116 (12.1) [10.2–14.4]	9.7 (7.3–12.8)	14.6 (11.6–18.2)	0.13	10.7 (8.7–13.1)	19.3 (13.6–26.6)	<0.001
Hispanic or Latino	143 (17.9) [15.4–20.7]	18.3 (14.7–22.5)	17.5 (14.1–21.5)		18.3 (15.5–21.5)	15.9 (10.8–22.8)	
White	641 (60.8) [57.5–63.9]	63.1 (58.5–67.5)	58.4 (53.8–62.8)		63.0 (59.5–66.4)	49.5 (41.8–57.1)	
Other race§	114 (9.2) [7.7–11.1]	8.9 (6.8–11.6)	9.5 (7.4–12.2)		8.0 (6.4–9.9)	15.4(10.8–21.4)	
Marital status				0.31			<0.001
Married or in a union	546 (52.4) [49.1–55.5]	53.7 (49.1–58.2)	51.0 (46.5–55.5)		51.5 (48.0–55.0)	56.6 (48.8–64.1)	
Divorced, widowed, or separated	179 (19.4) [16.9–22.1]	20.2 (16.7–24.3)	18.5 (15.1–22.3)		21.6 (18.7–24.7)	8.2 (4.8–13.4)	
Never married	289 (28.3) [25.5–31.3]	26.1 (22.3–30.2)	30.6 (26.6–34.9)		26.9 (23.9–30.1)	35.2 (28.2–43.0)	
Education				0.005			0.07
High school or less	327 (39.1) [35.9–42.4]	34.7 (30.3–39.4)	43.5 (39.0–48.1)		38.0 (34.5–41.6)	44.7 (37.1–52.5)	
Some college	265 (24.6) [22.0–27.4]	23.9 (20.3–27.8)	25.4 (21.7–29.5)		25.4 (22.6–28.6)	20.6 (15.3–27.2)	
Bachelor's degree	275 (23.8) [21.3–26.5]	27.9 (24.1–31.9)	19.7 (16.5–23.3)		24.8 (22.0–27.8)	18.9 (13.9–25.3)	
Postgraduate degree	147 (12.5) [10.6–14.6]	13.5 (10.9–16.6)	11.5 (9.0–14.4)		11.8 (9.9–14.1)	15.8 (11.2–21.7)	
Children in household				<0.001			0.05
Y	349 (31.2) [28.4–34.1]	25.7 (22.1–29.7)	36.7 (32.5–41.1)		29.9 (26.8–33.2)	37.5 (30.6–45.0)	
N	665 (68.8) [65.9–71.6]	74.3 (70.3–77.9)	63.3 (58.9–67.5)		70.1 (66.8–73.2)	62.5 (55.0–69.4)	
Household income, USD				0.02			0.39
<25,000	182 (20.4) [17.8–23.2]	17.1 (13.7–21.1)	23.7 (19.9–27.8)		20.2 (17.4–23.4)	21.0 (15.3–28.1)	
25,000– 49,999	266 (27.6) [24.8–30.6]	25.8 (22.0–30.1)	29.3 (25.3–33.7)		26.6 (23.6–29.9)	32.5 (25.6–40.2)	
50,000– 99,999	311 (29.3) [26.5–32.3]	31.6 (27.6–35.9)	26.9 (23.2–31.1)		30.1 (27.1–33.4)	25.1 (19.0–32.4)	
>100,000	255 (22.8) [20.3–25.5]	25.4 (21.8–29.5)	20.1 (16.8–23.7)		23.0 (20.3–26.0)	21.4 (16.1–28.0)	
Community type				<0.001			0.003
Urban	328 (31.7) [28.8–34.7]	26.7 (22.9–30.9)	36.6 (32.4–41.1)		29.4 (26.3–32.7)	43.1 (35.7–50.9)	
Suburban	463 (45.9) [42.7–49.1]	52.2 (47.7–56.7)	39.5 (35.1–44.0)		47.6 (44.1–51.1)	37.4 (30.3–45.2)	
Rural	223 (22.5) [19.9–25.3]	21.1 (17.6–25.0)	23.9 (20.2–28.0)		23.1 (20.2–26.2)	19.4 (14.1–26.2)	
Region¶				0.36			0.33
Northeast	182 (17.3) [15.0–19.8]	16.2 (13.1–19.8)	18.3 (15.2–22.0)		16.3 (13.9–19.1)	21.9 (16.3–28.8)	
Midwest	199 (20.5) [18.0–23.2]	20.6 (17.2–24.6)	20.3 (16.8–24.3)		21.0 (18.3–24.1)	17.6 (12.4–24.4)	
South	382 (38.6) [35.5–41.8]	37.2 (32.9–41.7)	40.0 (35.7–44.6)		38.6 (35.2–42.1)	38.6 (31.4–46.4)	
West	251 (23.7) [21.1–26.5]	26 (22.2–30.1)	21.3 (17.9–25.2)		24.0 (21.2–27.1)	21.9 (16.3–28.8)	

*Unweighted counts and weighted percentages. †Statistical significance of differences in responses was determined at α = 0.05 by using Pearson χ^2^ tests corrected for survey design. ‡Persons of Hispanic or Latino (Hispanic) origin might be of any race but are categorized as Hispanic; all racial group tabulation is limited to those persons reporting being non-Hispanic. §Includes Middle Eastern or North African persons, persons identifying as another race not listed in the survey, and persons identifying as >1 race. ¶Northeast: Connecticut, Maine, Massachusetts, New Hampshire, New Jersey, New York, Pennsylvania, Rhode Island, Vermont; Midwest: Iowa, Illinois, Indiana, Kansas, Michigan, Minnesota, Missouri, North Dakota, Nebraska, Ohio, South Dakota, Wisconsin; South: Alabama, Arkansas, District of Columbia, Delaware, Florida, Georgia, Kentucky, Louisiana, Maryland, Mississippi, North Carolina, Oklahoma, South Carolina, Tennessee, Texas, Virginia, West Virginia; West: Alaska, Arizona, California, Colorado, Hawaii, Idaho, Montana, Nevada, New Mexico, Oregon, Utah, Washington, Wyoming.

**Figure:**
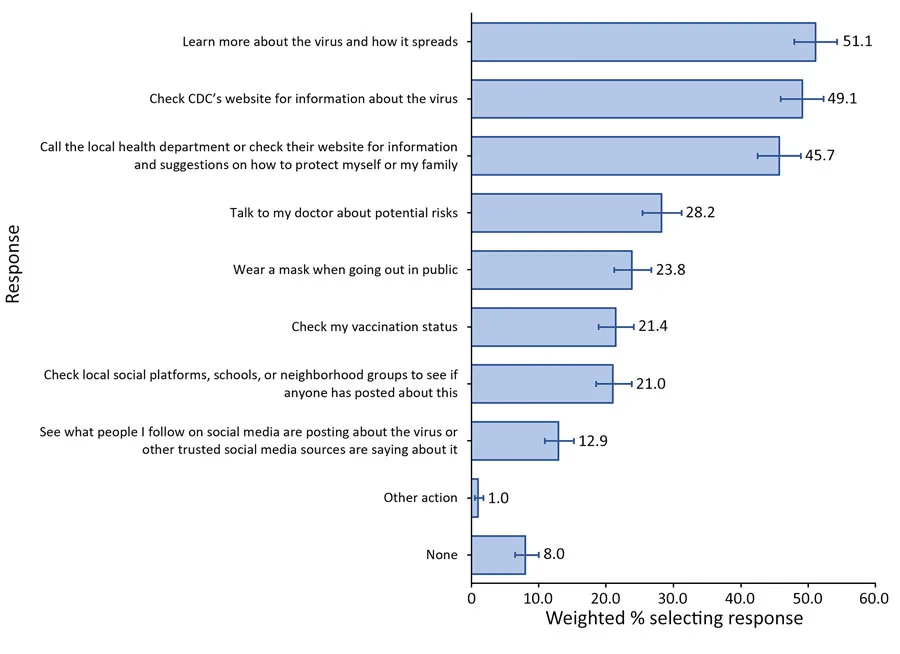
Actions participants intend to take after wastewater detections of a hypothetical vaccine-preventable virus in their area (n = 1,014) from Porter Novelli View survey, United States, August 2025. Bars indicate the percentage of participants who selected the action in their response to the question, “If wastewater data showed that this virus was present in your community, what steps, if any, would you take?” for which they could select multiple options; error bars indicate 95% CIs. CDC, Centers for Disease Control and Prevention.

Most (79.0%) participants gave free responses that suggested that information from WS about viruses and variants was important. Among the 801 participants who considered WS important, 624 (77.9%) wrote about early detection, outbreak prevention, awareness, and family or community safety. Of those, 79 (12.7%) participants specifically mentioned virus strains or vaccinations. One participant stated, “Wastewater monitoring helps detect which viruses are spreading and even identify their variants.” Another participant noted, “[WS] can tell you if the strain of the virus is a new one that you may not have been vaccinated against, and you can take the necessary precautions.” One participant suggested clear, nonalarming communication for informing the public. Among those who considered WS unimportant, reasons mentioned included uncertainty surrounding actions to take and beliefs that diseases will spread regardless of efforts.

In our survey, participants were moderately aware of WS, with approximately half of participants correctly defining it. That result was consistent with other nationwide surveys reporting awareness levels of 48% in 2022 and 49% in 2023 and a 2024 survey conducted across 5 states that reported 56.0% awareness (*8*,*9*). Furthermore, most participants accurately interpreted a figure depicting wastewater detection data, suggesting that an intuitive graphic can be helpful to convey information. When given background information on the types of infectious disease data WS provides to the public, participant attitudes were generally favorable.

To maximize data usability, health agencies could provide clear, plain-language explanations of WS accompanied by graphics, figures, and guidance for data interpretation for the pathogen. In addition, offering practical protective steps when pathogen detections occur might empower persons to make informed decisions.

Demographic differences in understanding might reflect varying levels of exposure to public health messaging and highlight opportunities for tailored communication (e.g., informing residents of rural communities of the value and use of WS). Study limitations include that persons with interest in public health or WS could have self-selected to participate in the survey and that the framing of the open-ended question might have led to measurement error in the form of social desirability bias (*10*). During times when WS detects increasing infectious disease activity, health agencies can partner with trusted messengers, including media partners, community organizations, and healthcare providers, who can help amplify and contextualize WS information for their communities and audiences. 

Appendix Additional information about public understanding of wastewater surveillance, United States, August 2025
