# Supplementary material for: Public Understanding of Wastewater Surveillance, United States, August 2025
Source: Emerg Infect Dis. 2026 Aug;32(Suppl 1):S111–4. doi: 10.3201/eid3213.260559 (PMC13585667; doi:10.3201/eid3213.260559)
Supplement: Supplementary file 1 [file 26-0559-Techapp-s1.pdf]

Article DOI: https://doi.org/10.3201/eid3213.260559

EID cannot ensure accessibility for supplementary materials supplied by authors. Readers who have difficulty accessing supplementary content should contact the authors for assistance.

Public Understanding of Wastewater Surveillance, United States, August 2025

Appendix

Methods

Porter Novelli Survey Methodology

This PN View survey was conducted by Big Village (https://big-village.com/insights/caravan-omnibus-surveys) among a sample of 1,014 U.S. adults 18 years of age and older. This survey was live on August 22–24, 2025.

PN View surveys are designed by Porter Novelli Public Services often with input from their clients. They are programmed and fielded by Big Village using opt-in panel members from Cint’s Insight Exchange (https://www.cint.com/esomar28). Quota sampling is used to collect a nationwide sample of respondents who are then weighted by sex, age, region, race/ethnicity, and education to mirror the demographical composition of the U.S. population using Current Population Survey proportions.

Data quality checks are incorporated during both sampling and survey administration. Cint uses a variety of tracking measures to confirm respondent identity and prevent duplicate responses. Big Village imbeds quality control questions within each survey to identify and remove inattentive respondents.

Although Porter Novelli Public Services and Big Village are not subject to institutional review board review, they adhere to professional standards and codes of conduct set forth by ESOMAR (the European Society for Opinion and Marketing Research, https://esomar.org/codes-guidelines) and the Insights Association (https://www.insightsassociation.org/About-Us/Code-of-Standards). Respondents are informed that their answers are being used for market research

and can refuse to answer any question at any time. No personal identifiers are included in the resulting data files.

Respondents for each survey are selected from among those who have volunteered to participate in online polls. They may be subject to multiple sources of error, including, but not limited to sampling error, coverage error, error associated with nonresponse, error associated with question wording and response options, and post-survey weighting and adjustments.

Traditional response rates cannot be calculated because response links are made available to anyone in the study sample pool. To ensure rapid fielding, no effort is made to limit the number of “clicks” so quotas often fill before respondents have a chance to respond.

Quantitative analysis

Participant characteristics are described overall and by ability to correctly define wastewater surveillance and interpret wastewater detection data visualization. Statistical difference was determined at $\alpha = 0.05$ using Pearson chi-square tests corrected for survey design.

Survey Instrument

1. When you hear the phrase ‘wastewater monitoring’ or ‘wastewater surveillance’, what do you think it means? Select the option that best matches your understanding. **[Select one answer. Randomize a-d]**

a. Checking for diseases that you can catch from being around wastewater (sewage)

b. Testing the sewage that goes into a treatment plant for infectious diseases that may be present in the community

c. Testing drinking water in your community for chlorine or other chemical levels

d. Testing different sources of standing water (i.e., ponds, pools, buckets, or open containers) for mosquitoes that can spread diseases

e. Other **[Please specify]**

f. I don’t know

Wastewater monitoring tests sewage at treatment plants for viruses circulating in the community. Results show which viruses are present, such as flu, and whether levels are rising or

falling. This helps public health officials track infections and inform communities, often before people develop symptoms.

2. How often would you like to receive updates about the levels of viruses found in your community's wastewater? **[Select one answer]**

a. I'd like regular updates (like weekly summaries)

b. I'd only want alerts when levels are high in my community

c. I'd want both regular updates and alerts for high levels in my community

d. I'm not sure how often I'd like to receive updates

e. I don't want to receive updates about the levels of viruses found in my community's wastewater.

3. How would you like to receive this information? **[Select as many as apply for a-f. Randomize a-e]**

a. Social media updates (i.e., Instagram, Facebook)

b. Weekly email newsletters

c. Community-based apps (i.e., Nextdoor, Citizen)

d. Hear about it on the news, radio, or podcasts

e. Text alerts

f. Other **[Please specify]**

g. I have no preference **[EXCLUSIVE]**

4. What sources would you trust to provide you with information about levels of viruses in your community's wastewater? **[Select as many as apply for a-i Randomize a-h]**

a. Friends, family, or co-workers

b. Social media influencers

c. State/Local public health department

d. CDC (Centers for Disease Control and Prevention)

e. Community organizations/nonprofits

f. Medical professionals

g. Local news channel

h. Local utility or water department

i. Other **[Please specify]**

j. I don't trust any source **[EXCLUSIVE]**

The following questions show how wastewater detections of a virus could appear on a CDC's Web site. For these questions, assume the virus spreads through coughing or sneezing, is vaccine-preventable, and people who are unvaccinated are at higher risk of getting sick.
[Programming note: allow 'Click to enlarge']

5. This hypothetical map of New Jersey and the surrounding area shows detections of the virus in wastewater for the last week (Appendix Figure). According to the map, how many detections of the virus were there in the past week? **[Select one answer]**

a. One detection

b. Two detections

c. 3–4 detections

d. 5–7 detections

e. 8–9 detections

f. 10–14 detections

g. Fifteen or more detections

h. I don't know

6. If wastewater data showed that this virus was present in your community, what steps, if any, would you take? **[Select as many as apply for a-i. Randomize a-h]**

a. Learn more about the virus and how it spreads

b. Check my vaccination status

c. Talk to my doctor about potential risks

d. Check local social platforms, schools, or neighborhood groups to see if anyone has posted about this

e. See what people I follow on social media are posting about the virus or other trusted social media sources are saying about it

f. Check CDC's Web site for information about the virus

g. Call the local health department or check their Web site for information and suggestions on how to protect myself/my family

h. Wear a mask when going out in public

i. Other **[Please specify]**

j. I would not take any action based on wastewater data **[EXCLUSIVE]**

7. Wastewater monitoring can show which viruses (like flu) are spreading in your area. It can also detect specific versions (or variants) of those viruses by analyzing their genetic makeup. Do you think this kind of information is important? Why or why not? **[Please type in your response]**

Results

Appendix Table. Public health communication preferences and trusted sources of information on community wastewater virus levels (N = 1,014) — Porter Novelli View survey, United States, August 2025

Public health communication preferences	No.	(%)	[95% CI]*
Preferred frequency to receive wastewater virus level updates†			
Receive alerts only when levels are high in the community	334	(31.9)	[29.0–34.9]
Regular updates (e.g., weekly summaries)	277	(27.8)	[25.0–30.8]
Receive both regular updates and alerts for high levels in the community	198	(19.4)	[17.0–22.1]
Not sure	104	(10.8)	[8.9–13.0]
Does not want to receive updates about the levels of viruses found in their community's wastewater	101	(10.1)	[8.3–12.3]
Preferred mode of delivery†			
Text alerts	407	(40.2)	[37.0–43.3]
News, radio, or podcasts	306	(29.8)	[27.0–32.8]
Weekly e-mail newsletters	298	(29.0)	[26.2–31.9]
Social media updates (i.e., Instagram, Facebook)	270	(26.0)	[23.3–28.9]
Community-based apps (i.e., Nextdoor, Citizen)	183	(17.2)	[14.9–19.7]
No preference	61	(6.3)	[4.9–8.1]
Does not want to receive updates about the levels of viruses found in their community's wastewater	101	(10.1)	[8.3–12.3]
Trusted source of information on community wastewater virus levels†			
State or local health department	572	(56.5)	[53.2–59.6]
Local utility or water department	519	(51.4)	[48.2–54.6]
Centers for Disease Control and Prevention	447	(43.6)	[40.5–46.8]
Local news channel	389	(38.1)	[35.0–41.3]
Medical professionals	338	(32.4)	[29.4–35.4]
Community organizations or non-profits	230	(21.4)	[19.0–24.1]
Friends, family, or co-workers	196	(18.4)	[16.1–21.0]
Social media influencers	86	(7.8)	[6.3–9.6]
None	54	(5.7)	[4.3–7.4]

*Unweighted counts and weighted percentages. Weighted percentages were computed using a denominator of 1,014 participants.
†Percentages may not add to 100% as participants could select more than one option.

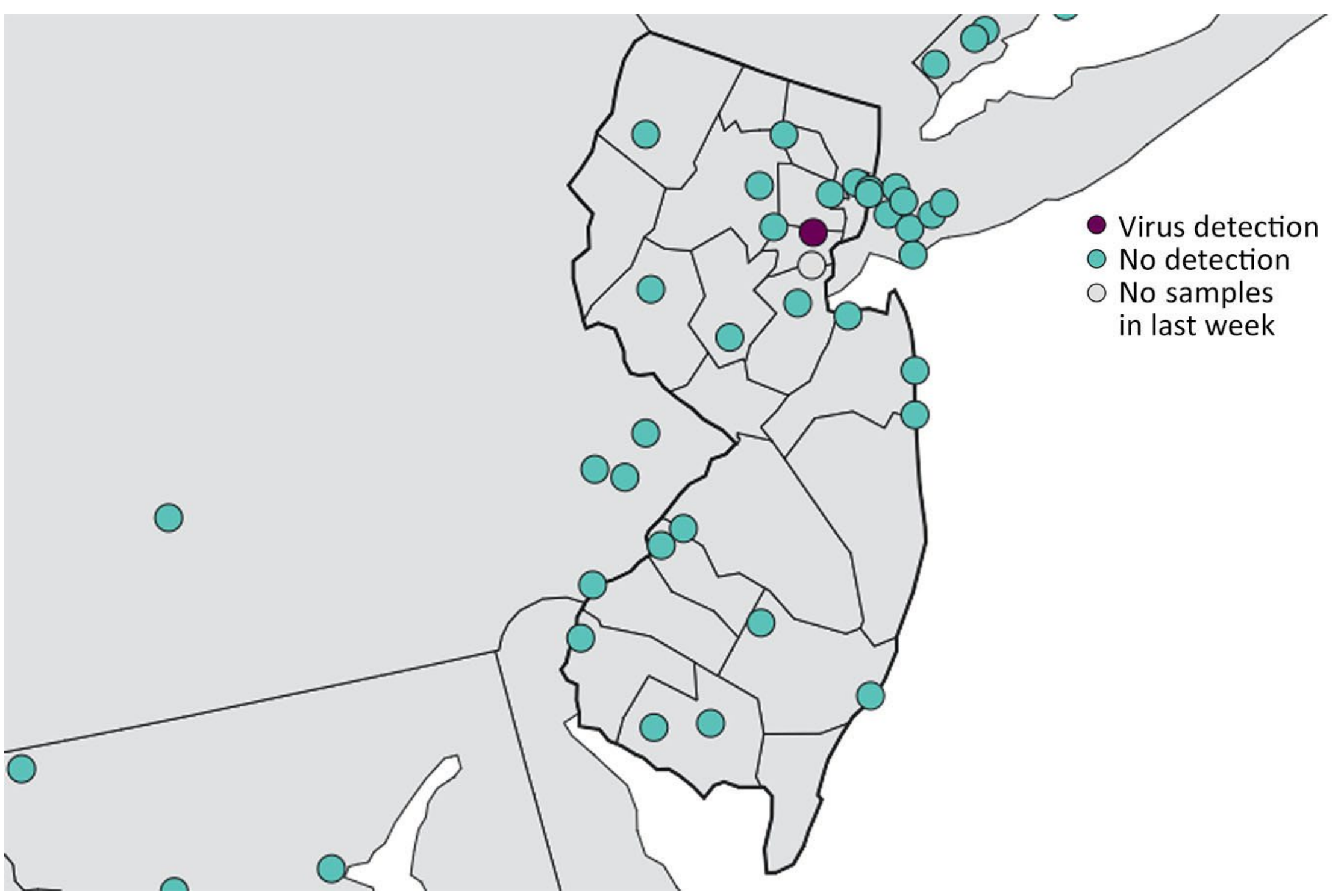

Appendix Figure. Hypothetical map of New Jersey, USA and surrounding areas.
